# Optimizing Safe Approaches to Manage Plant-Parasitic Nematodes

**DOI:** 10.3390/plants10091911

**Published:** 2021-09-14

**Authors:** Mahfouz M. M. Abd-Elgawad

**Affiliations:** Plant Pathology Department, National Research Centre, El-Behooth St., Dokki, Giza 12622, Egypt; mahfouzian2000@yahoo.com

**Keywords:** nematode management, biological control, mechanisms, host plant resistance, synthetic nematicide, botanicals, optimizing strategies

## Abstract

Plant-parasitic nematodes (PPNs) infect and cause substantial yield losses of many foods, feed, and fiber crops. Increasing concern over chemical nematicides has increased interest in safe alternative methods to minimize these losses. This review focuses on the use and potential of current methods such as biologicals, botanicals, non-host crops, and related rotations, as well as modern techniques against PPNs in sustainable agroecosystems. To evaluate their potential for control, this review offers overviews of their interactions with other biotic and abiotic factors from the standpoint of PPN management. The positive or negative roles of specific production practices are assessed in the context of integrated pest management. Examples are given to reinforce PPN control and increase crop yields via dual-purpose, sequential, and co-application of agricultural inputs. The involved PPN control mechanisms were reviewed with suggestions to optimize their gains. Using the biologicals would preferably be backed by agricultural conservation practices to face issues related to their reliability, inconsistency, and slow activity against PPNs. These practices may comprise offering supplementary resources, such as adequate organic matter, enhancing their habitat quality via specific soil amendments, and reducing or avoiding negative influences of pesticides. Soil microbiome and planted genotypes should be manipulated in specific nematode-suppressive soils to conserve native biologicals that serve to control PPNs. Culture-dependent techniques may be expanded to use promising microbial groups of the suppressive soils to recycle in their host populations. Other modern techniques for PPN control are discussed to maximize their efficient use.

## 1. Introduction

Plant-parasitic nematodes (PPNs) can cause significant losses in the size and quality of a wide range of economically important crops. Previously, regulatory and sanitation measures entirely avoided their casual introduction, minimized their spread, and/or reduced their damage. However, the current widespread and severe damage of PPNs lead to the need for additional control measures. Synthetic nematicides have shown some nematode control with consequent yield increase—but many of them have been restricted or banned. This is due to their adverse effects on human health and the environment, as well as damage to the durability of many agricultural ecosystems. Increasing concern over such chemical nematicides has led to unprecedented and great efforts in various research areas to manage these pests safely and effectively.

Current nematode research has addressed genetics and molecular patterns associated with plant defense and damage in the event of nematode infection [1]. Research has also addressed microbial priming [2], which has achieved tremendous progress. Various techniques are being developed to fully grasp the interaction between PPNs and their host and non-host plants via the elicitor-receptor reciprocal action [3,4]. These substantial mechanisms are expected to provide us with the needed information to design durable nematode resistance in plants. Moreover, the processes engaged in plant defense and protection against PPN can be activated by beneficial microbes and synthetic elicitors that can be soundly and effectively exploited [4].

Various aspects of the current research focus on the fundamentals of the PPN–plant relationship. However, there have been related opportunities to exploit the available applications to safely control nematodes. Thus, benign alternative methods to chemical nematicides are expected to make up many of the the durable crop protection strategies. Abd-Elgawad [5] recently addressed the general strategies of using safe antagonists of PPNs. They are generally based on either augmentation (inundative and inoculative) or conservation biological control. This review extends and updates implementations of such strategies. It highlights the use and potential of various strategies and tactics that can contribute to PPN management. Such approaches may include biological control agents (BCAs), the use of botanicals (e.g., antagonistic plants), host plant resistance to nematodes with related crop rotations, and other advanced treatments. The desired outcome is not only to avoid plant damage and yield losses caused by the nematodes and contribute as best we can in sustainable agricultural ecosystems, but to summarize current progress made in the research and application of these techniques. It also presents key factors affecting their success and broader exploitation, as well as their merits and demerits, and discusses agricultural practices that optimize PPN control.

## 2. Biological Control Agents 

### 2.1. Their General Categorization and Effects

Fungal and bacterial organisms are currently considered the most efficient and major biocontrol agents (BCAs) against PPNs [6,7]. Others, such as predaceous nematodes, mites, viruses, protozoans, oligochaetes, collembola, algae, and turbellarians, are also BCAs, but are less effective and less studied. Given the common occurrence of such numerous BCAs and their bioactive compounds, it is uncertain whether they can routinely limit PPN populations. To test their benefits, many hindrances must be overcome, including their mass culture, formulation, application techniques, and interactions, as they are applied to the seed, cultivated soil, or seedling medium for PPN control. The modes of action of these BCAs may be categorized into two major groups, i.e., either direct antagonism against PPNs or indirectly promoting operators of plant growth. However, BCAs and/or their bioactive metabolites are responsible for PPN management via various mechanisms. For instance, these fungi may be endoparasitic, toxin-producing, nematode-trapping, and/or parasites of eggs, juveniles, or adults. The other main taxon of BCAs was recently reviewed and shown to contain endophytic bacteria, rhizobacteria, obligate parasitic bacteria, symbiotic bacteria, opportunistic parasitic bacteria, and cry protein-forming bacteria [5]. These BCAs can also produce plant growth promotors for plant growth [8]. Directly, they can assist plants by easing resource possession and production of active compounds and hormones (e.g., gibberellins and cytokinin) necessary for plant growth. Indirectly, they can produce lytic enzymes and antibiotics to suppress pests and pathogens. These BCAs can also prime plants for PPN resistance. Molinari and Leonetti [2] have recently reported that BCAs can interact with roots to prime plants against infection by root-knot nematodes (RKNs), *Meloidogyne* spp., via upregulation of endogenous defense genes. They may comprise salicylic acid-dependent pathogenesis-related genes of the systemic acquired resistance, such as PR-1, PR-1b, PR-3, and PR-5. Moreover, related enzymes, e.g., endochitinase and glucanase, showed elevated activities in roots of pre-treated inoculated plants, which may open new avenues to novel PPN control. 

### 2.2. Fungal and Bacterial Biocontrol

Fungal species related to genera, such as *Trichoderma*, *Purpureocillium*, *Catenaria*, *Actylellina*, *Dactylellina*, *Arthrobotrys*, *Aspergillus*, *Monacrosporium*, *Hirsutella*, and *Pochonia*, are outstanding BCAs against PPNs, especially for RKN control [6,9,10]. For example, endophytic fungi of the genera *Trichoderma*, *Fusarium*, *Alternaria*, *Purpureocillium*, and *Acremonium* can colonize plant roots and enhance plant defense via multiple factors [11]. They may repel RKN second-stage juveniles (J_2_) away from roots, retard or attenuate PPN development, or lower their fecundity. Species of *Trichoderma* and *Purpureocillium* can kill RKNs at different life stages in the root systems or soil. *Pochonia chlamydosporia* can induce systemic resistance against *M*. *incognita.* They can also target other important PPNs, such as the cyst nematodes, *Globodera* spp., especially when combined with additional BCA [7,12]. Silva S. et al. [13] screened 33 strains of *P*. *chlamydosporia* and *Purpureocillium lilacinum* and selected the most promising ones, e.g., *P*. *lilacinum* (CG1042, CG1101) and *P*. *chlamydosporia* (CG1006, CG1044) to be tested against *Meloidogyne enterolobii*, a relatively recently described but important RKN species, especially for tomato and banana. Both *P*. *lilacinum* and *P. chlamydosporia* caused 44 and 34% suppression in *M*. *enterolobii* eggs on tomato roots, respectively, whereas 34% suppression in *M*. *enterolobii* eggs was recorded on banana roots by *P. chlamydosporia*. However, such efficacies were noted when inoculation level of *M*. *enterolobii* eggs was as low as 500 eggs. Thus, applying both species, within the context of integrated pest management (IPM) programs, is suggested when *M*. *enterolobii* population levels are low. Another group of potential fungi is the arbuscular mycorrhizal fungi (AMF), which function as obligate plant root symbionts. The plant offers photosynthetic carbon for the symbionts, while the latter assist roots to uptake higher nutrients and boost both root growth and structure. Moreover, they usually compete for nutrition and space with PPNs and induce plant systemic resistance [14]. 

Likewise, numerous bacterial species of many genera, such as *Pseudomonas*, *Serratia*, *Bacillus*, *Pasteuria*, *Achromobacter*, *Variovorax*, *Rhizobium*, *Agrobacterium*, *Comamonas*, *Arthrobacter*, and *Burkholderia*, have shown nematicidal activities against PPNs [15,16,17,18]. Their modes of action against PPNs may vary even within a genus [7], but generally comprise antagonism, antibiotic production, and/or induced resistance. For instance, their nematicidal operations against RKNs are based on toxic particles of cry proteins in *Bacillus thuringiensis*, toxic antibiotics in *B*. *subtilis* and *B*. *cereus*, enzymatic activity in *B*. *firmus*, and repelling RKN J_2_ by *B*. *cereus* after colonizing the roots. Strain efficacy may be more specific; the *B*. cereus strain BCM2 could reduce *M*. *incognita* invasion on tomato roots by 67.1% relative to the control. The *B*. *cereus* strain that colonizes tomato roots could affect the root exudates by raising the secretion of certain repellent *M. incognita* J_2_ substances [19]. Lee and Kim [20] found that chitinase and protease, produced by *B*. *pumilus* strain L1, were responsible for *M*. *arenaria* antagonistic traits. They induced about 90% J_2_ mortality and 88% inhibition of egg hatch.

The obligate parasites, *Pasteuria* spp., are extremely safe BCAs to manage PPNs. They can act on the nematodes under tough ecological conditions and with variable soil temperature, pH, and moisture. Their spores usually attach to the cuticle surface of the specific nematode species/race as they move about in the soil. Once adhered, they set up germ tubes that break into the nematode’s interior body. The internal proliferation of these cells and sporulation suppresses nematode multiplication and causes nematode mortality. As they are species-specific, *Pasteuria* spp. do not hurt non-target organisms, e.g., as a RKN-specific parasite, *P. penetrans* can only infect the related J_2_. The attached spores restrict the nematode movement and make them stick to the nearby nematodes. If the PPN can mature, the female may produce a few or no eggs in host plants. Abd-Elgawad [5] reviewed the attributes which allow *Pasteuria* spp. to integrate with other safe approaches, e.g., crop rotation, soil amendments, and nematode-resistant cultivars, to manage PPNs. Their endospores are resistant to mechanical shearing, drying, and heat. However, *Pasteuria* isolates should be screened to select the most adequate one(s) for biocontrol in specific agroecosystems because they are very specific and may only attack certain isolates of a given species.

### 2.3. Nematode-Suppressive Soils

Suppressive soils were reviewed as those in which harmful pathogens and parasites, herein PPNs, cannot set up or persist, found, but lead to no disease, or become established and initiate a mild disease that soon recedes [21]. The biological activity of such a specific soil is documented when its suppressiveness: (1) is removed by biocides; (2) can be conveyed to conducive soil with a modest volume of suppressive soil; (3) is specific to a nematode species; (4) can reduce multiplication in root-knot and cyst nematodes in the root zone; (5) can be detected by baiting methods; (6) is heat sensitive; (7) is density-dependent. To achieve these attributes, the BCAs in nematode-suppressive soils can act directly as nematode antagonists and/or they can indirectly prime plants and induce their defense responses against PPNs. Antibiosis and parasitism by BCAs were also suggested in a few soils with specific PPN suppressiveness. Topalović et al. [10] appraised fungi and bacteria that were characterized in PPN-suppressive soils via next-generation sequencing or extracted from dead or diseased PPNs. They noted that soil suppression may act against the relevant PPN species as the microbiome may vary from one soil to another. For instance, suppressive soil was more efficient in *M*. *hapla* than the *M. incognita* control. Additionally, soil properties and plant species/cultivar can also influence the magnitude of this suppression. Thus, to avoid the impact of soil physicochemical and nutritional features, Topalovic et al. [9] altered the approach of transferring soil suppressiveness to the conducive soil by using the microbial component of the suppressive soil only in a water suspension. However, the soilless suspension could not cause mortality of any PPN species, but it could in tomato-planted soil with the suspension [9]. 

The magnitudes of root colonization by BCAs and their possible metabolites and induced resistance are impacted by plant genotype. Nematode-susceptible plants will harbor more PPNs and need more BCAs to suppress them than poor host plants. Although two isolates of *P. chlamydosporia* prompted systemic resistance against RKNs, the induction was plant species-dependent. This reduced *M. incognita* female fecundity, infection, and reproduction of tomatoes, but not cucumbers [21]. Moreover, in a separate monoculture of different sugar beet cultivars in *Heterodera schachti*-infested soil, *H*. *schachtii*-tolerant cv. “Pauletta” enabled suppressiveness to be set up without the initial yield decrease noted in susceptible cv. “Beretta” [22]. Botelho et al. [23] speculated that the biological and physicochemical attributes of the coffee rhizosphere could dictate their impact on *Meloidogyne exigua* suppression under field conditions. Thus, such suppressive soils caused about 83% *M*. *exigua* J_2_ mortality and attained the highest yields of coffee beans. Thus, further plant–nematode–microbe interactions in suppressive soils require additional study to be better understood to enable novel insights for the best exploitation of suppressiveness. Westphal [5] reported a few methods to examine the biology of PPN soil suppressiveness. They mostly rely on comparing PPN reproduction in sterilized vs. non-sterilized soils. A drawback in this approach is that the growth parameters of plants are usually better in sterilized soil, which impacts the PPN activities and other biologicals as well. Therefore, it may bias the results [24]. While culture-independent methods on the related microbiome have given a better understanding of the functional potential of many PPN suppressive BCAs, culture-dependent techniques enabled the use of some microbial groups in specific suppressive soils [10]. Both approaches should be timely and adequately used to adjust recycling of the relevant microbiome in their host populations and expanded long-term PPN suppression in other soils.

### 2.4. Evaluating Factors Affecting Their Success

#### 2.4.1. Biological and Ecological Factors

Many factors can affect BCA–nematode interactions. Hence, the biology and ecology of these BCAs should be grasped from the standpoint of pest management so that they can be properly harnessed in biocontrol. Currently, facilitated-omics techniques should contribute to the better holistic perception of biocontrol mechanisms with related colonization processes at the rhizosphere and the relevant factors influencing them. In this vein, Cámara-Almirón et al. [25] reviewed the molecular basis used by many bacteria to antagonize plant pathogens and enhance plant growth and the structural units, necessary for their biofilm setting, e.g., the formation of the bacterial biofilm often influences and ensures a stable and effective biocontrol. 

Basically, the biological strain, dose, time, and application method that likely achieve the best BCA(s)–nematode host matching should be optimized for the desired level of PPN management. The best BCA that properly fits the properties of the targeted habitats, cultivated crops, and PPNs to optimize the biocontrol gains should be selected. These factors can modify, to varying degrees, the effects of BCAs on the PPN control programs. Therefore, there is often a gap between BCA efficacy in the field and controlled experimental conditions. Careful manipulation of these factors can improve the effects of BCAs, especially in terms of their being more inconsistent, less effective, and/or slower-acting than pest control via chemicals. Admittedly, as soil food webs, including PPNs, have a cryptic nature, BCA ecology and their role in modifying nematode population levels and dynamics are largely unknown and deserve further study. Hence, significant variables can provide insights into how soil properties can be modulated to enhance biocontrol by conserving and favoring specific settings or BCAs [9,10,22,26,27,28]. For example, soil moisture and texture [29], salinity [30], mulching [31], and pH [32] were found to modulate nematode populations directly or indirectly by influencing their hosts or enemies [33].

Manufacturers of biocontrol products must consider these factors and their outputs [5]. Otherwise, a biocontrol product may contain several genera or groups of BCAs to increase its potency. Still, only one BCA in another product may be more effective than this combination (e.g., NemOut^®^ (contains Bacillus subtilis + B. licheniformis + Trichoderma longibrachiatum) vs. Rizotec^®^ (contains only *Pochonia chlamydosporia*)) [13].

Using BCAs is not an easy or routine task, but should be based on accurate, complementary data and a good conception of the possible involved factors. Therefore, sampling and primary tests are prerequisites to obtain the data on the related factors for effective IPM. Biological suppression may be assessed by comparing nematode reproduction in both untreated and treated soils with a proper biocide or heat to eliminate BCAs [24]. This test may take 1–3 months as targeted reproduction of PPNs is valued after at least one nematode generation. To shorten this period, survival of only free-living stages of the concerned PPNs may be assessed after several days in the untreated and treated soils. This alternative test offers rapid conclusions but, as such, is limited to measuring the effect of only BCAs on soil or migratory stages of PPNs. In the latter tests, other BCAs specialized in parasitizing PPN eggs and/or nematode-sedentary stages are mistakenly ignored. Furthermore, a PPN species not present in the field soil is preferably utilized in both tests to determine the level of biosuppression to avert the confusing impact of native nematodes, e.g., a host-specific parasite cannot be avoided. For instance, using the reniform nematode, *Rotylenchulus reniformis*, to evaluate its biosuppression in the sting nematode *Belonolaimus longicaudatus*-infested soil cannot detect a species-specific BCA, e.g., *Candidatus Pasteuria* usage, which is specific to parasitizing *B. longicaudatus* [34]. Mixing a small amount of the field soil into disinfested soil may resolve the issue. In this case, *B. longicaudatus* is conveyed with the field soil, and so other target PPNs can be added to the soil with few influences. Notwithstanding the utility of endemic *B. longicaudatus* to detect species-specific antagonists, the BCAs conveyed with the soil should have enough time to multiply to suppressive levels. The test does not negate that biocontrol of PPNs using an introduced BCA may not be as effective in various settings as that of indigenous BCA, due to ecological validity [35]. Eventually, relevant bioassays that validate PPN suppression in a specific agroecosystem should be carried out for the best BCA–PPN host matching.

#### 2.4.2. Agricultural Practices

The positive or negative role of specific production practices should be assessed preferably in the context of IPM programs. Clearly, regulatory and phytosanitary measures should be exercised to avoid PPN contamination of plant materials, cultivated media, and used equipment [36,37]. Generally, fertilization and soil amendments can boost plant growth with consequent possible increases in population levels of both plant parasites and BCAs. However, significant exceptions should be considered [35]. Whenever possible, they must be manipulated to set up an adequate soil environment to boost and/or protect BCAs for conservation biological control. This is important for stable agroecosystems, e.g., alfalfa, turfgrass, and perennial crops, that can likely favor this conservation for long-term persistence of, especially indigenous, BCAs. In contrast, intensive production practices, soil infestations by polyspecific nematodes, introducing seedlings infected with new PPNs, and short crop sequences or sequential susceptible host plants may decrease the occurrence and persistence of these BCAs.

It is important not only to recognize promising BCAs, but also to combine them with conservation operations in the planted soil to enhance the efficacy, consistency, and duration of BCAs. These operations should manipulate the soil habitat to benefit the introduced BCA and any other useful organisms at the expense of PPNs. *Streptomyces*-treated soil showed a reduction in the total bacterial population that significantly changed the rhizosphere microbiome. However, the *Streptomyces* population in the treated rhizosphere was enhanced with less RKN damage to tomato roots [38]. Thus, the practices may comprise offering extra resources to BCAs, enhancing their habitat quality, and reducing or avoiding negative influences of pesticides on them. Resources, such as adequate organic matter, may be added to elevate the existing BCA efficacy and persistence. Specific soil amendments can boost the quality of particular soils [27]. On the contrary, pesticide usage, tillage, crop rotation, and fallow periods can directly disrupt BCA populations. Surprisingly, BCAs may be adversely affected if these practices can decrease their PPN hosts. Thus, these practices should also be adjusted for the effective use of the introduced BCAs in case of augmentation (inundative or inoculative) biological control. More selective pesticides and careful spatial (horizontal and vertical) and temporal usage of nematicides should be followed to evade or at least lessen their damage to BCAs [39].

The use of BCAs can also be optimized via sequential application or co-application with compatible agricultural inputs. The co-usage of *P*. *chlamydosporia* and chitosan enabled more root colonization by the fungi and less RKN damage than either alone [40]. Abd-Elgawad and Askary [7] reviewed effective strategies that combine BCAs with other cultural inputs to operate additively or synergistically in IPM. Optimistically, more recent operations are still being explored with other microbes. Admittedly, one of the present pressing issues is related to how to adequately address developing multi-dimensional biocontrol programs. One trend is to simultaneously use closely related or compatible species of BCAs that can offer optimum biocontrol efficacy, domination in the soil microbiome, and/or better root protection. Combining *B*. *subtilis* with *B*. *pumilus* gave more average increments in growth parameters of *M*. *incognita*-infected cowpea plants than either alone [41]. Thus, it is likely that different *Bacillus* species may present unlike anti-RKN mechanisms, which open new avenues to improve the biocontrol efficacy. Another trend is sequential usage. Dahlin et al. [42] found that fluopyram (a nematicide) reduced the *M*. *incognita* population on tomatoes at planting and that adding the *P*. *lilacinum* strain PL251 during the growing season could reinforce the reduction. The reductions in the number of *M*. *incognita*-J_2_ were 56 and 68% when PL251 was applied alone and after fluopyram, respectively. Fewer *M*. *incognita* galls were found as *B. firmus* preceded synthetic nematicides in consecutive tomato cycles. The galling severity assessed by the galling index (scale of 0–10) was reduced from 7.7 in the control roots to 5.9, 4.3, and 4.0 for *B*. *firmus*, *B*. *firmus* + oxamyl, and *B. firmus* + fosthiazate, respectively [43]. These favorable results should be extended and documented as BCAs are compatible with other chemicals.

Dual-purpose BCAs should be utilized against more than one group of crop pests whenever possible. For instance, three populations of entomopathogenic nematodes, used as biocontrol agents against insect pests, could significantly (*p* < 0.05) reduce *M*. *incognita* populations on watermelon at 7 and 21 days after treatments [44]. Moreover, the entomopatogenic fungus *Lecanicillium muscarium* isolates (Lm1, Lm2, Lm3) were effective against *M*. *incognita* on tomatoes [45]. The isolate Lm1 reduced *M*. *incognita* egg and gall numbers by 90 and 80%, respectively. Thus, in these cases, it is assumed that BCAs could control both insect pests and PPNs. Sharma and Sharma [46] demonstrated suppression in various *M*. *incognita* stages and reproduction on tomato roots caused by individual or dual inoculations of AMF and plant growth-promoting rhizobacteria (PGPR). They found that colonization of AMF (*Rhizophagus irregularis*) and PGPR (*Pseudomonas jessenii* strain R62 and *Pseudomonas synxantha* strain R81) induced tomato resistance against RKN by upregulating the defense genes. 

Nonetheless, it is suggested herein to examine the effects of BCAs on a case-by-case basis. This will allow us to monitor and optimize various techniques of PPN control according to the existing variables. It would enable us to unravel the complexity of the factors governing the activity and reproduction of PPNs and their associated BCAs in general or specific suppressive soils. In a soil microbiome, factors that positively affect specific BCAs that repel, suppress, or kill PPNs via competition, predation, or parasitism should be utilized for better plant growth. Likewise, adding amendments or related materials to the soil to release compounds that are toxic to the plant pathogens and/or inducing the defense systems of the host plants should be thoroughly examined and correctly adjusted [35,36]. Eventually, adopted recommendations to combat agricultural pests by decision makers and governmental bodies should guide and enlighten growers for optimizing practical use of bionematicides—especially in developing countries such as Egypt [47].

## 3. Botanicals as Bionematicides

### 3.1. Antagonistic Cultivated Plants 

During their growth, antagonistic cultivated plants produce antihelminthic compounds that act as antagonists to the nematodes via various modes of action [48]. The nematostatic or nematicide compounds in the plant organs may be freed into the soil or operate within the plant to act as nematode traps or show unfavorable responses to PPNs. The broad conception of these plants may include different groups that can adversely affect various PPN populations, but poor and non-hosts will better be addressed separately [36] hereafter in more detail. 

Many antagonistic plant species are found, but the most famous ones that are utilized against important PPNs include *Tagetes* spp., *Azadirahta indica*, *Brassica* spp., and *Crotalaria* spp. [48]. Various species of the genus *Tagetes* (marigold) may reduce PPN populations via different modes of action, e.g., by acting as a poor host or non-host, generate allelopathic compounds, trap the nematodes, or induce nematode antagonistic flora/fauna. Derivatives of bithienyl and alpha-terthienyl produced by marigold are toxic to the nematodes [49]. Species of marigold, such as *T. patula, T. erecta*, and *T. minuta*, are efficient, especially against two nematode genera, *Meloidogyne* and *Pratylenchus* [48]. Contrary to a tomato–tomato rotation, *T*. *erecta*, *T*. *patula*, and *T*. *signata* decreased RKN galling in subsequent susceptible tomato plants. Although the neem (*Azadirahta indica*) tree is generally considered to be antagonistic to many pests, the neem-based products are most commonly used in PPN control. They could demonstrate good nematicidal activities. Moreover, many species of *Brassica* (cruciferous plants), such as cabbage, broccoli, rape, canola, and mustard, can form glucosinolates (GSLs). Hydrolysis of secondary metabolite GSLs results in volatile and toxic isothiocynates (ITCs), which act as biofumigants against PPNs. Thus, the nematicidal properties of ITCs released into the soil may be attributed to aromatic GSLs (roots), indole GSLs (root and shoot), and aliphatic GSLs (seeds) when these repositories are rotated with PPN-susceptible plant species or grown as cover crops [50]. The biofumigation range for PPN control has been widened to include non-brassica antagonistic species. They can also form volatile pathogen-suppressing molecules. The hydrolysis of antecedent cyanogenic glycoside/dhurrin in the graminaceous plants (e.g., sudangrass (*Sorghum* × *drummondi*) and sorghum (*Sorghum bicolor*)) may produce cyanides that can kill PPNs [27]. Sunn hemp (*Crotalaria juncea*) as a cover crop and green manure is commonly utilized for its antagonistic impacts on RKNs in numerous crops. Moreover, *C*. *longirostrata* is is incorporated into the soil after growing as a cover crop to decrease RKN galling. Its effect concerning PPN control may be due to toxins produced during microbial degradation, not by toxic exudates from the plant [36]. Other species were tested for PPN control activity [51]. *Pratylenchus brachyurus* reproduction rates were lowered when maize (*Zea mays*) was intercropped or rotated with bristle oats and oilseed radish [52]. Growth of maize was enhanced (up to 34%) when intercropped with velvet bean (*Mucuna pruriens*) or jack bean (*Canavalia ensiformis*), while *Pratylenchus zeae* population levels were suppressed by 32%. Yields of maize intercropped with jack bean were raised (22–190%) under field conditions [51]. 

### 3.2. Plant-Related Materials and Compounds

Using the relevant compounds via extraction from the plants or incorporating plant parts into the soil is another and more common tactic for PPN control than using the entire plants. These materials are mainly extracted or formed from antagonistic plants. They may be grouped under various terms, such as natural compounds, organic acids, essential oils (EOs), and plant extracts and compounds. In contrast, not all of these groups are exclusively related to plants. For instance, acetic acid is produced as secondary plant metabolites [53] or as culture filtrates of the bacterium, *Lactobacillus brevis*, strain WiKim0069 [54]. This acid can damage the cuticle of RKN J_2_, vacuolize the cytoplasm, and degrade the nuclei, causing death [54]. Numerous organic acids, such as amino, propionic, formic, and butyric acids, can exert toxic effects on PPN species [18]. They are formed via microbial decomposition of other compounds in the soil, mostly those related to plant materials/residues, but may also result from metabolites formed by soil organisms. Others, such as sesquiterpene heptalic acid produced by the fungus, *Trichoderma viride* [7], and hydroxamic acids from the grass, *Secale cereale* [55], have proved effective against important PPN species.

Neem’s natural compounds, such as azadirachtin, kaempferol, thionemone, nimbidin, quercetin, nimbin, and salannin, also have nematicidal properties. Intercropped or treated plant roots, via soil application, can absorb these materials. Moreover, many natural nematicidal compounds have drawn the attention of the pesticide industry to develop their extraction and related processes. Hence, other effective treatments of neem may comprise root dipping in neem leaf extracts, soil amendment with its leaf extracts, mulching the soil with dried or fresh leaves, seed coating or drenching the soil with neem extract or oil, application of root exudates, or treating soil with seed or kernel powder [56]. Botanical extracts have conspicuous merits over synthetic nematicides. For example, they contain new compounds for PPN management [18,48]. Thus, nematodes are not yet able to develop resistance or inactivate them. In addition to originating from natural resources and having rapid biodegradation, they are always less concentrated and thus always less toxic than pure materials. These traits support their use as ecologically benign alternative nematicides.

Moreover, EOs have been tested for PPN control. The differences are clear in their related efficacies. Abd-Elgawad and Omer [57] tested the EOs of four medicinal plant species in the family Lamiaceae for PPN control. *Mentba spicata* caused the highest PPN mortality, followed by *Thymus vulgaris, Majorana hortensis*, and then *Mentha longifolia.* The corresponding major oils in these plants were carvone, P-cymene, terpinen-4-ol, and carvone, respectively. Generally, 0.1 oil solutions of each plant could inhibit more than 80% of *M. incognita* J_2_ relative to 3.5% at the untreated check. Among the PPNs tested, *Rotylenchulus reniformis* was better controlled by the oil solutions than nematode species related to the genera *Criconemella* and *Hoplolaimus*. Recently, only 14 out of 29 EOs could have nematicidal efficacy in the range 8–100% at 1000 μg/mL, whereas the EO of Mexican tea (*Dysphania ambrosioides*) was the most efficient [58]. Mexican tea EO eliminated 99.5 and 100% of galls and eggs on susceptible tomato plants, respectively. They found that p-cymene (3.35%), E-ascaridole (8.45%), and (Z)-ascaridole (87.28%) formed 99.08% of the total composition in *D*. *ambrosioides* oil. 

Kalaiselvi et al. [59] found that the elevation at which the plants are cultivated may affect their EOs in terms of quantity, chemical composition, nature, yield, and appearance. When *Artemisia nilagrica* was grown in low and high lands, its extracted EOs showed different lethal concentrations (LCs) against *M*. *incognita* on tomato. The LC_50_/48 h was 10.23 and 5.75 μg/mL for EOs of low- and high-altitude plants, respectively. The EOs of low- and high-level plants decreased *M*. *incognita* (J_2_ and eggs) per 10 g root by approximately 68 and 87%, respectively. These oils also enhanced plant growth differently. 

Many studies tested plant extracts against PPNs. Recent focus has been placed upon their compounds [9,10,60,61] as they are generally less toxic and safe alternatives to synthetic chemicals that can also efficiently control PPNs. A plant extract may have both acid and oil together, which can enhance nematode mortality as in vetiver grass *Vetiveria zizanioides* [62]. Extracts of *Paenoia rockii* and *Camellia oleifera* could inhibit egg hatching and J_2_ activity of *M*. *incognita* [63]. Nematicidal activity of aqueous and methanolic extracts of *Ricinus communis*, *Taxus baccata*, *Raphanus raphanistrum*, *Sinapis arvensis*, and *Peganum harmala* on *M*. *incognita* J_2_s was assessed at various exposure times and doses. Methanolic extracts showed more influence than the aqueous ones. Moreover, methanolic extracts of *S. arvensis*, *P. harmala*, and *T. baccata* had peak RKN mortality—86.6, 89.2, and 100%, respectively [60]. 

### 3.3. Safety, Reliability, and Economics of the Related Nematicidal Products

Some botanical-based nematicides are being commercially marketed, while others are still in the pipeline. For instance, numerous effective neem-based nematicidal formulations have been marketed, such as Neemrich, Neemix, Neemazal, Neemgold, and Neemax. In contrast, Nemastop is a commercial product with garlic (*Allium sativum*) extract (600 g ground garlic cloves/1 water). This has been marketed for PPN control but is not as effective as synthetic chemical nematicides or even other commercial biocontrol agents on eggplant [64]. However, this does not negate PPN suppression by allicin (diallyl thiosulfinate), the effective nematicidal compound of garlic. Allicin could control *M*. *incognita* and improve tomato yield [65]. Host range claims of bionematicidal products are often taken from the manufacturer’s product labels. They have not necessarily been confirmed in neutral trials [66]. Under favorable conditions, PPN control usually increases with consequently elevated crop yield as a product concentration and/or exposure time is also enhanced. 

Three basic elements are required for the bionematicides, in general, to be successful: (1) safety to the environment and human health, (2) reliable nematicidal effect, and (3) favorable economics. For instance, among synthetic chemical nematicides, ITCs are included as active ingredients. Notwithstanding the utility of natural ITCs as biofumigants against PPNs, they may share the same biochemical mechanism of action against the targeted PPNs. Thus, negative effects of ITCs as in mustard biofumigants have caused vulnerability and instability of soil food webs and suppression of beneficial organisms [27,67]. Ntalli and Caboni [50] speculated that non-target organisms are also adversely affected because both synthetic and natural components of ITCs interact in a non-specific and irreversible manner with amino acids and proteins. Hence, more studies harnessing their safe utilization as integrants in pest management programs should be conducted. On the contrary, azadirachtin compounds are relatively safe pesticides relevant to ecological issues and environmental risk. They have faint potential mobility in soil and degrade rapidly. Azadirachtin is non-mutagenic and pure azadirachtin is non-toxic to humans. Additionally, it possesses relative selectivity. Thus, it is safe for beneficial insects and can be utilized in IPM programs [48]. *Tagetes* spp. compounds in soil need to be further examined to establish their fate or degradation periods in field situations. On the one hand, researchers and stakeholders should consider that the notion of safety is relative and should be quantified as these materials are still chemicals, but not synthetic ones. On the other hand, several plant materials/extracts have been synthesized to ensure more safety for human application than the commonly known synthetic chemical nematicides.

Sikora et al. [51] suggested that antagonistic plants are very attractive tools for PPN control, but there are potentially new ones that could also be identified. Moreover, techniques should be sought for efficient and multi-purpose applications. Other merits of antagonistic plants are their effective operation in upgrading the soil characteristics. They are used as organic matter and green cover to raise soil quality [68]. Specific groups of antagonistic plants may possess additional merits. A striking example is to boost the activity of biocontrol agents against PPN in addition to their direct effect of reducing damage from pests. Contrary to the bacteria associated with soybean roots, the rhizobacteria isolated from the roots of antagonistic plant species *Ricinus communis*, *Mucuna deeringiana*, and *Canavalia ensiformis* could significantly decrease both *Meloidogyne incognita* and *Heterodera glycines* population densities on roots of soybean plants. Hence, Grubišić et al. [48] speculated that these plants may retain a selective action within each pest class as they possess multiple mechanisms with a wide spectrum. Additionally, these antagonists, related to legumes, can fix the atmospheric nitrogen, which boosts soil fertility. 

More research is direly needed to determine the optimum conditions for these bionematicides in general. To optimize PPN control, their incorporation into the soil should target nematode-life stages and species that are most vulnerable. Variables, such as edaphic factors, tillage systems, proper planting date, favorable plant species, and suitable growth stage, should be examined to be best tailored for PPN control. Notwithstanding the nematicidal activity of brassicas cover crops to suppress PPN populations in soil, they may not provide consistent efficacy. Dutta et al. [27] stressed that temperatures may be too high for such plants to adequately show their nematicidal activities under greenhouse conditions. 

A study of economic feasibility for applying any of the botanicals to PPN control should be conducted. If economic factors are not convenient, even a strategy involving good nematicidal properties to the targeted PPNs is doomed to recede. Components of economic success comprise the grower’s sense to avoid crop losses caused by the nematode pests, the relative expenses of using this bionematicide compared to other options for PPN control, the value of the commodity (e.g., per acre), and its price in the relevant market. Thus, a grower should be enlightened about the indirect benefits of these safe nematicides, e.g., their use to avoid ecological pollution and health hazards, as well as to avoid nematode resistance of chemical nematicides. Such an agricultural extension would encourage growers to use them. Other policies may lower costs and improve the success of specific botanicals. Marigold seeds, for example, are costly relative to seeds of cover crops because marigold has a high value as an ornamental plant. Therefore, Grubišić et al. [48] suggested that the expenses of its seeds would be reasonably priced, or even lowered, if they were commercialized on a large scale as cover cropping for PPN management programs. 

## 4. Exploiting Poor- or Non-Host Crops

As there are many nematode-susceptible plant species, we should make full use of poor- or non-host genotypes [69,70,71]. These are species/cultivars with genotypes that are immune, resistant, or tolerant to one or more of the PPN species. They are marketed based on their yield—not as cover crops utilized for pasturage or soil amendments/conservation [36]. Resistant or non-host plants can contribute to solving many PPN-related issues. Ensuring adequate crop sequences using non-host crops is the most effective method utilized for global RKN management. Such immune or resistant plant cultivars can be employed to enhance crop yields, suppress PPN population levels, and boost effective crop rotations [36]. Nematode-tolerant cultivars are usually called non-hosts—although PPN can reproduce on them. However, they can withstand nematode attack, and their crop yields are not significantly affected [71]. They can often enable less nematode reproduction than susceptible cultivars [22]. 

Older, related conceptions must be updated. Resistance-breaking pathotypes or populations of a nematode species may be able to reproduce on a cultivar that is known to be resistant to this species. These virulent populations can emerge in the field via repeated exposure to the resistance genes in the plants and may adversely affect resistance durability. However, such populations usually show faint competitiveness and less reproductive capacity on susceptible hosts than wild populations. In addition, virulent pathotypes reproduce only on plants with the gene on which their selection happened [72]. 

In a soil having a polyspecific nematode community, a non-host cultivar for one species may also be the susceptible host for another species. Fortunately, reasonable rotation crops for important PPN species with their host range size are listed [36]. It is justifiable to test any of the listed non-hosts in the targeted soil before recommending them. Moreover, the non-host cultivars would preferably be used in integrated PPN management strategies to avoid continuous resistant cultivar cultivation. As resistance-breaking PPN pathotypes may seriously result from this continuity, they can degrade sustainable crop production systems. Moreover, resistance found in some tomato cultivars failed, due to heat instability or sensitivity of the resistant *Mi* gene to high temperature. Incremental reductions in RKN resistance started as the soil temperature was raised above 25.6 °C. Thus, at 32.8 °C, resistance to RKN infection in these plants was fully broken [73]. Hence, such cultivars may be restricted to regions or seasons with cool soil temperatures. 

Resistant cultivars of major crops, such as RKN-resistant tomato, are globally available, but emerging technical and economic issues with releasing others should be resolved [74]. Traditional techniques for host suitability designations of serious PPN species have been reviewed [74,75,76], but biochemical and DNA markers have merits to complement their phenotype screens [2,77]. Crucial factors affecting the phenotypic expression of the resistance should be adequately examined. Thus, a multi-disciplinary approach combining plant breeders, molecular biologists, and nematologists should investigate the level, nature, and inheritance of resistance traits. They should explore any DNA recombination during breeding cycles. Biochemical markers of genetic traits for PPN resistance should be sought in genome-assisted breeding strategies for their introgression into elite cultivars. 

## 5. Other Methods of PPN Management

### 5.1. Additional Soil Amendments and Treatments 

The broad concept of soil amendments is to use not only plant materials as a cover crop, compost, seed meal, and green manure, but also to mix them with other components. Contrary to the above-mentioned botanicals, they may include, for instance, various animal manures and/or nutrient salts to form different varieties of amendments. These additions are mostly organic matter and have been used in multi-purpose agricultural practices. They can suppress the population levels of many pathogens, pests, and weeds, enrich soil fertility, boost soil structure, increase communities of beneficial organisms, and/or induce systemic resistance of plant species [27]. Organic amendment herein refers to organic material brought from outside to the inside of the soil, e.g., industrial waste products or processing residues. This differs from the above-mentioned botanicals, which were added as fresh crop residue or grown in the rotation, e.g., break, cover, trap, antagonistic or green manure crops. Usually, merging large amounts of such organic material into the soil will reduce PPN densities. These may include many materials, such as oil cakes, sawdust, coffee husks, crustacean skeletons, chicken manure, paper waste, and crop residues, which showed various degrees of PPN control [36]. This action was mostly associated with corresponding increases in crop yields. 

Moreover, an amendment that works well in soil with specific edaphic and biological factors may not work at all in another soil. Optimizing the PPN control efficacy relies on its compounds’ compositions, quality, and quantity of its associated and interacting microbiome, and its ability to break down these compounds into elements that are suitable for plant growth and/or harmful to the nematodes. Fresh compost enriched with beneficial organisms and nutrients may show better efficacy against PPNs than aged compost. Abiotic factors, such as soil moisture and temperature, usually influence the microbiome and decomposition of these compounds. Soil amended with chicken manure and broccoli at ≥25 °C was superior to the same at 20 °C in reducing *M. incognita* galls on tomato roots [78]. Ntalli et al. [79] reviewed various soil amendments and their specific nematicidal activities. They categorized amendments as Brassicaceae and Asteraceae species (for cover-crop, biofumigation, rotation, and incorporation), biochars, composts, and vermicomposts (applied as recycling wastes), and other self-made products, such as canola or orange peel meals, dried leaves of *Canabis sativa*, and marigold or pennycress seed powder. Examples of safe strategies for applying various bionematicides or biocontrol methods against important nematode species are given (Table 1). Nonetheless, some examples may need continuous improvement to the above-mentioned aspects to improve their efficacy and reliability. 

Using various composts as big sources of soil amendments should be further exploited. They could be manipulated via fermentation processes to make them enriched in the desired microbial species and PPN antagonistic compounds, such as phenolics and humic acids [85]. Composts can also enhance soil resident microbial antagonists, boost plant resistance or tolerance to various stresses, such as PPN infection, and change soil profiles to improper media for PPN reproduction. These gains should be optimized to improve PPN control by grasping the related edaphic factors as well. Eventually, their processes and components should be employed to obtain the desired PPN-suppressive soils.

Other treatments may be used when relevant factors and economic feasibility permit. For high cash crops, heating the soil can effectively manage RKN in protected cultivation [86]. Soil solarization could be effective against PPNs. Tarping the soil surface, especially in sunny regions, with transparent plastic sheets will raise soil temperatures enough to kill many pests and pathogens [87]. Solarization is more effective against PPNs in contained raised beds for cultivation in warm regions. Kokalis-Burelle et al. [88] found that the number of RKN galls on roots of sunflowers, snapdragon, and larkspur were less in steam-treated soil than in solarization alone. Steam treatment was as effective as methyl bromide in controlling *M*. *arenaria*. They concluded that soil steaming followed by solarization is so effective that it can be a safe alternative to chemical nematicides [88]. 

Biodisinfestation or biosolarization, that is, using soil amendments before solarization, could enhance the pest and pathogen suppression via rapid generation of harmful compounds, such as acetic and butyric acids, ultraviolet radiation, and lack of oxygen, due to microbial anaerobiosis [27]. However, lethal temperature and related duration may vary from one pathogen species to another [87]. Thus, sustained low PPN populations were sometimes not affected by fairly high temperatures (≤45 °C). In these cases, lasting PPN populations at deeper depths away from the sun could recolonize and infect the plant roots. 

Ozonated water, O_3_wat, was reported to control *M*. *incognita* likely via modulated antioxidant systems without phytotoxicity. Tomato plants treated with O_3_wat after or before *M*. *incognita* inoculation showed a root galling index (on a scale of 0–10) of 1.9 or 1.6, respectively, compared to 3.9 in the check [89]. As it degrades to water in a short time, O_3_wat could suppress RKN populations early in the growing season without adverse effects.

### 5.2. Advanced Methods

**Figure 1:**
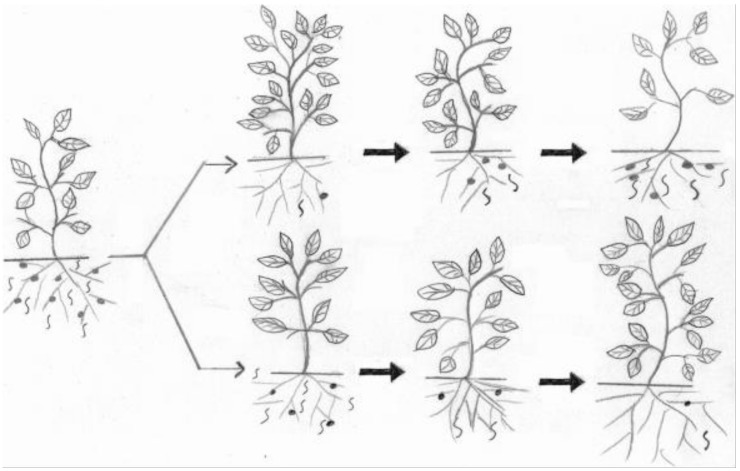
Effect of a chemical nematicide (upper trend) and a bionematicide (lower trend) on root-knot nematodes on susceptible plants. When both nematicides were applied, the chemical has a rapid and significant effect, reducing the nematode population. However, a few nematodes can escape its effect and reproduce to reach a damaging level, while the bionematicide can work continuously to keep the nematode below the economic threshold level [91].

Our targeted agroecosystems are facing real challenges that require advanced methods and innovative thinking for safe and effective PPN control. Some of the biggest challenges are the increased banning of numerous effective but synthetic chemical nematicides, vertical and horizontal agricultural expansion to raise and improve food production, the frequent appearance of resistance-breaking nematode pathotypes, global warming backing rapid PPN reproduction and spread and discovery of new PPN species [26] (to name but a few related to aggravated nematode damage). Hence, new PPN management tactics and strategies should detect more resilient BCAs and related materials that can best match these expected ecological windows of the pests and pathogens [90]. For instance, efficient methods for better understanding biological and ecological factors that affect BCAs should be employed [91]. This will enable bionematicides to effectively replace unsafe chemical nematicides for sustainable agriculture (Figure 1). Moreover, specific wavelengths via near-infrared spectroscopy could be used to detect soil nematode collections with different functions assigned to definite sets of soil organic matter [92]. Furthermore, developing bioactive compounds that have natural multifunctional derivatives, including nematicidal activity, are in progress. One such derivative is the chitin oligosaccharide dithicyclobutane (COSDTB) derivative. The 1, 3-dithicyclobutane-N-chitosan oligosaccharide could decrease *M*. *incognita* egg hatching by up to 90% at 2 mg/mL and cause 94% mortality of *M*. *incognita* J_2_ at 4 mg/mL [93]. The role of silicon to support plant resistance against a variety of harmful bacterial and fungal invasions was recently reviewed [94]. As its salts can also suppress *Meloidogyne paranaensis* populations on coffee seedlings [95], silicates should be further tried in IPM programs in specific sites with various groups of pathogens. Formulating industrial wastes as value-added products for PPN control will also optimize the gaining of the related industries. Waste such as orange bagasse, soybean hull, rice husk, poultry litter, and common bean hull were assessed for *M*. *javanica* control in the glasshouse [96]. Their mixtures, orange bagasse, soybean hulls, and powdered bean hulls, had a range of 55–100% RKN control. Other promising BCAs or their metabolites against PPNs are still under experimentation, e.g., *Rhodoblastus acidophilus* strain PSB-01 [97] and *Mortierella globalpina* [98]. On the other hand, nanoparticles [99] have proved to possess promising physical and chemical characteristics against nematodes. They have demonstrated effective PPN control with a few possible demerits.

Although optimizing PPN sampling and extraction methods to avoid their misuse and achieve cost-effective and efficient IPM programs are recently emphasized [100], such advances for PPN control should biotechnologically keep up in parallel to these improvements. The current research on genome sequencing technologies, small interfering RNA techniques (RNAi), and targeted genome editing should be harnessed to better grasp plant–nematode interaction mechanisms, and molecular enhancing of PPN-plant resistance should be used to boost these programs [1]. 

Likewise, other approaches may include expanding targeted biological seed treatment, remote sensing for specific nematicide applications, screening quarantine regulations, minimum tillage to potentiate PPN antagonists, biochemical marker-orientated selection for plant resistance, molecular monitoring and detection of PPNs, and indexing of PPN biodiversity via metagenomics [36]. Their expansion should not negate the continuous search for finding new BCA isolates or genetically engineered ones that are more persistent and compatible with beneficial rhizosphere organisms. There are application techniques that have not been tested on a large scale in earnest to develop them, e.g., spraying BCAs around the base of plants, practical use of a slow-release system, or dipping root plugs into BCA suspensions. Systematic experimentations and field trials testing the aforementioned techniques in various settings to show their worth with feasible, economical insights must be a way forward in crop protection/pest management.

Numerous biological products have demonstrated promising BCA efficacies against PPNs with low costs relative to other chemical nematicides. Table 2 lists examples of such costs for both types of products with their application rates as authorized by the Egyptian Ministry of Agriculture [47]. Thus, it is possible that using the BCA-listed products can offer control comparable to but more economical than these synthetic chemicals (Table 2). Hence, growers should be familiar with these products and their optimized usage, as well as the above-mentioned relevant agricultural practices via agricultural extensions. The end in view is that these bionematicides will ensure more safe applications, while promoting crop yields.

## 6. Conclusions

The current literature on using safe approaches to manage PPNs is extensive given the considerable and negative effects of the synthetic chemical nematicides. These approaches may use various tactics and strategies, including different materials, such as BCAs, botanicals, poor- or non-host crops, and other advanced methods. However, such benign techniques mostly need to be further developed and/or optimized as many BCAs, for example, are more inconsistent, less effective, and/or slower-acting in nematode control than synthetic chemicals. Therefore, agricultural practices that favor the conservation biocontrol of PPNs should be recognized and earnestly applied. Moreover, bionematicides can be included in IPM programs in various ways that make them complementary or superior to these chemicals; they can exert synergistic or additive effects with other agricultural inputs. As numerous bionematicides are or are likely to become widely available soon, seeking their optimal performance is a continuous process. Hence, research priorities for harnessing such relevant and advanced methods should be identified to boost soil fertility within sustainable agricultural production systems. This will necessitate grasping the complex network of interactions among biotic and abiotic factors in intimate contact with these bionematicides to maximize their gains. Thus, the biology and ecology of these bionematicides can be seen as a research priority; they may even need to use previously developed, sophisticated methodologies. Meanwhile, stakeholders, such as nematologists and agronomists, can train, assist, and guide extension officers and farmers to optimize the quality of their produce. This can be achieved by minimizing the adverse effect of the pests in their crops via the improved and efficient application efficacy of these bionematicides.

## Figures and Tables

**Table 1:** Examples of various biocontrol agents and strategies against important nematode species.

Biological Control Agent	Nematode Species	Type of Study	Host Plant	Reference
Bacteria				
*Bacillus firmus*	*Meloidogyne incognita*	In vivo	tomato	[43]
*Pasteuria penetranse*	*Meloidogyne exigua*	In vivo	coffee	[23]
*PGPR: Pseudomonas jessenii* and *P. synxantha*	*M. incognita*	In vivo	tomato	[46]
Fungi				
(A) Filamentous: *Trichoderma* spp.	*Rotylenchulus reniformis*, *M*. *javanica*, *M*. *incognita*, *Heterodera cajani*	In vivo	tomato, brinjal, okra, soybean, sugarbeet, pigeonpea	[7]
AMF: *Rhizophagus irregularis*	*M. incognita*	In vivo	tomato	[46]
Endophyte: *Fusarium oxysporum*	*Radopholus similis*	In vivo	banana	[80]
(B) Mushrooms: *Lentinula edodes, Macrocybe titans, Pleurotus eryngii*	*M*. *javanica*	*In vitro*	tomato	[81]
(C) Yeasts: *Saccharomyces cerevisiae*	*M. incognita*	In vivo	eggplant	[82]
Co-application: *Pochonia chlamydosporia* and Chitosan	*M. javanica*	In vivo	tomato	[40]
Sequential application: Fluopyram and *Purpureocillium lilacinum*	*M. incognita*	In vivo	tomato	[42]
Dual-purpose: *Heterorhabditis bacteriophora* EGG	*M. incognita*	In vivo	watermelon	[44]
Algae: *Chlorella vulgaris*	*M. incognita*	In planta	potato	[83]
Nematode-suppressive soil	*M. hapla*, *Pratylenchus neglectus*	In vivo	tomato	[9]
Botanicals: *Tagetes* spp.	*M. incognita*, *M*. *javanica*, *M. acrita*	In vivo	tomato and *eggplant*	[48]
Soil amendments	*M. incognita*, *Heterodera glycines*	In vivo	tomato and soybean	[79]
RNA interference via stimulants of soil streptomycetes	*Heterodera avenae*	In planta	wheat	[84]

**Table 2:** Examples of bionematicidal product costs and used rates as compared to chemical nematicides in Egypt.

Active Ingredient	Product Name	Application Rate (Product Hectare ^−1^) ^+^	Price per Hectare
Abamectin (soluble concentrate at 20 g/L) generated from the fermentation process of *Streptomyces avermitilis*	Tervigo 2% SC	5.95 L/Hectare	USD 319
10^9^ CFU/mL of *Serratia* sp., *Pseudomonas* sp., *Azotobacter* sp., *Bacillus circulans* and *B. thuringiensis*	Micronema	71.4 L/Hectare (thrice)/year	USD 40
10^8^ units/mL *Purpureocillium lilacinus*	Bio-Nematon	4.76 L/Hectare/year	USD 78
10^9^ bacterium cells of *Serratia marcescens*/mL water	Nemaless	23.8 L/Hectare (thrice)/year	USD 95
Cadusafos (O-ethyl S,S-bis (1-methylpropyl) phosphorodithioate)	Rugby 10 G	57.14 Kg/Hectare	USD 1028
Oxamyl (methyl 2-(dimethylamino)-*N*-(methylcarbamoyloxy)-2 oxoethanimidothioate)	Vydate 24% SL	9.52 L/Hectare (twice)/year	USD 445

^+^ The rates are evenly applied to the soil (except oxamyl for foliar application). In some cases, these rates may be incorporated into potting mix, field soil, or applied in greenhouses for which other doses may be used according to the manufacturer’s product labels on different crops [47,101,102,103,104].
